# A comparative analysis of image harmonization techniques in mitigating differences in CT acquisition and reconstruction

**DOI:** 10.1088/1361-6560/adabad

**Published:** 2025-02-27

**Authors:** Anil Yadav, Spencer Welland, John M Hoffman, Grace Hyun J Kim, Matthew S Brown, Ashley E Prosper, Denise R Aberle, Michael F McNitt-Gray, William Hsu

**Affiliations:** 1 Department of Bioengineering, Samueli School of Engineering, University of California, Los Angeles, CA 90095, United States of America; 2 Medical & Imaging Informatics Group, Department of Radiological Sciences, David Geffen School of Medicine at UCLA, Los Angeles, CA 90095, United States of America; 3 Center for Computer Vision and Imaging Biomarkers, Department of Radiological Sciences, David Geffen School of Medicine at UCLA, Los Angeles, CA 90095, United States of America

**Keywords:** Computed Tomography, Image Reconstruction, Harmonization, Convolutional Neural Network, Generative Adversarial Network

## Abstract

*Objective*. The study aims to systematically characterize the effect of CT parameter variations on images and lung radiomic and deep features, and to evaluate the ability of different image harmonization methods to mitigate the observed variations. *Approach*. A retrospective in-house sinogram dataset of 100 low-dose chest CT scans was reconstructed by varying radiation dose (100%, 25%, 10%) and reconstruction kernels (smooth, medium, sharp). A set of image processing, convolutional neural network (CNNs), and generative adversarial network-based (GANs) methods were trained to harmonize all image conditions to a reference condition (100% dose, medium kernel). Harmonized scans were evaluated for image similarity using peak signal-to-noise ratio (PSNR), structural similarity index measure (SSIM), and learned perceptual image patch similarity (LPIPS), and for the reproducibility of radiomic and deep features using concordance correlation coefficient (CCC). *Main Results*. CNNs consistently yielded higher image similarity metrics amongst others; for Sharp/10%, which exhibited the poorest visual similarity, PSNR increased from a mean ± CI of 17.763 ± 0.492 to 31.925 ± 0.571, SSIM from 0.219 ± 0.009 to 0.754 ± 0.017, and LPIPS decreased from 0.490 ± 0.005 to 0.275 ± 0.016. Texture-based radiomic features exhibited a greater degree of variability across conditions, i.e. a CCC of 0.500 ± 0.332, compared to intensity-based features (0.972 ± 0.045). GANs achieved the highest CCC (0.969 ± 0.009 for radiomic and 0.841 ± 0.070 for deep features) amongst others. CNNs are suitable if downstream applications necessitate visual interpretation of images, whereas GANs are better alternatives for generating reproducible quantitative image features needed for machine learning applications. *Significance*. Understanding the efficacy of harmonization in addressing multi-parameter variability is crucial for optimizing diagnostic accuracy and a critical step toward building generalizable models suitable for clinical use.

## 1. Introduction

The growing availability of clinical CT scans has led to a proliferation of studies exploring the use of quantitative image features extracted from CT scans in applications such as diagnosis, prognosis, and treatment selection (Lambin *et al* 2017, Thawani *et al* 2018). While many studies show the predictive value of radiomic and deep features, and their potential to assist with tasks such as lesion detection, characterization, and quantification (Aerts *et al* 2014, Sala *et al* 2017), few models that incorporate these features have successfully been translated into clinical practice.

One contributing factor, among many others, is the variation in CT scans that occurs due to differences in dose and reconstruction kernel, which adversely affect downstream analyses (Emaminejad *et al* 2018, Orlhac *et al* 2019). For instance, in lung cancer screening, small regions of high contrast and subtle texture changes are associated with the presence of a nodule. However, differences in CT parameters can change the nodule appearance on CT scans and features extracted from them, which can further undermine the reliability of machine learning model outputs (Li *et al* 2018, Kim *et al* 2019, Park *et al* 2019).

Harmonization of reconstructed image data is one approach to minimize scanner-specific effects and achieve consistent downstream task performance across different reconstructions. Mitigating the effects of radiation dose has been the focus of multiple prior studies involving low-dose CT (LDCT) (Hu *et al* 2023). Chen *et al* used a residual encoder-decoder convolutional neural network (CNN) to remove noise in CT (2021), while Li *et al* leveraged the cyclic nature of generative adversarial networks (GANs) in combination with a Wassertian GAN framework to improve LDCT images (2021). Super-resolution techniques have been used to address image resolution differences. Park *et al* used a U-net to overcome partial volume effects by learning an end-to-end mapping between 15 mm and 3 mm slice thickness images (2018). Lee *et al* (2019) used a CNN to convert scans between smooth and sharp kernels and Choe *et al* (2019) investigated the impact of reconstruction kernels on radiomic feature reproducibility.

Krishnan *et al* used a conditional Pix2Pix-based GAN approach for harmonizing reconstruction kernel differences in lung CT scans, leading to significant improvements in radiomic feature reproducibility while enhancing consistency in quantitative emphysema and body composition measurements (2024). Recent works have explored transformer and diffusion-based models for improving CT image quality. Wang *et al* proposed a transformer architecture with a Token2Token dilated block to enhance feature representation in LDCT denoising with lower computational overhead (2023). Zhang *et al* used a lightweight convolutional encoder combined with transformer blocks and an efficient patch-based self-attention module to improve noise suppression, structure preservation, and lesion detection (2023). Karageorgos *et al* applied a denoising diffusion probabilistic model to enhance the diagnostic quality of CT scans affected by metal artifacts (2024).

While various techniques have been published to mitigate the effects of individual CT parameters on image quality, the impact of systematic variation across multiple CT parameters on the efficacy of harmonization methods remains underexplored. The current paradigm for selecting a harmonization technique is largely unstructured and lacks a standardized approach. One notable effort presents a checklist for choosing data harmonization strategies by considering factors such as the availability of computational resources, data type, modality, and scale (Nan *et al* 2022). However, this approach does not account for the complex interactions between multiple CT parameters and their combined impact on harmonization outcomes across different downstream analyses, leaving a significant gap in understanding the challenges posed by multi-parameter variability for harmonization methods.

We present a comparative analysis that considers how the image was acquired and the downstream analysis to characterize the effect of image harmonization in mitigating variations in image acquisition and reconstruction parameters. The contributions of this study are two-fold: (1) we demonstrate how CT parameters (simulated radiation dose and reconstruction kernel) affect image similarity and reproducibility of quantitative image features, and (2) we assess the efficacy of different categories of harmonization techniques in mitigating the effects of differences in these parameters. We examine the results of each evaluation metric under different imaging conditions to determine whether harmonization is necessary under specific image parameters and pinpoint scenarios that could benefit from harmonization, along with the preferred technique to achieve the best performance for each downstream analysis. This study establishes a systematic approach for selecting the optimal harmonization method by considering the relationship between CT parameters, harmonization techniques, and the downstream analysis.

## 2. Methods

### 2.1. Dataset

This study investigates the impact of two CT parameters (simulated radiation dose and reconstruction kernel) using a retrospective in-house dataset of 100 low-dose chest CT (LDCT) exams of lung cancer screening-eligible patients (e.g. aged 50–80, with a 20 pack-year smoking history, and currently smoking or having quit within the past 15 years). The data was collected under IRB/ethics board protocol 11-000 126 (Computer Analysis of CT Images) using a Siemens Definition AS64 scanner, following a low-dose lung cancer screening approach (120 kV, 25 mAs, pitch 1.0, 0.5 s rotation, 64 × 0.6 mm collimation) with reconstructions at 1 mm thickness and interval, utilizing the B45 kernel. This resulted in a CTDIvol of ∼2 mGy for a standard-sized patient. As described in Hoffman *et al* (2019), Poisson noise was introduced to the raw projection data at levels that were equivalent to 10% and 25% of the original dose.

**Figure 1.**
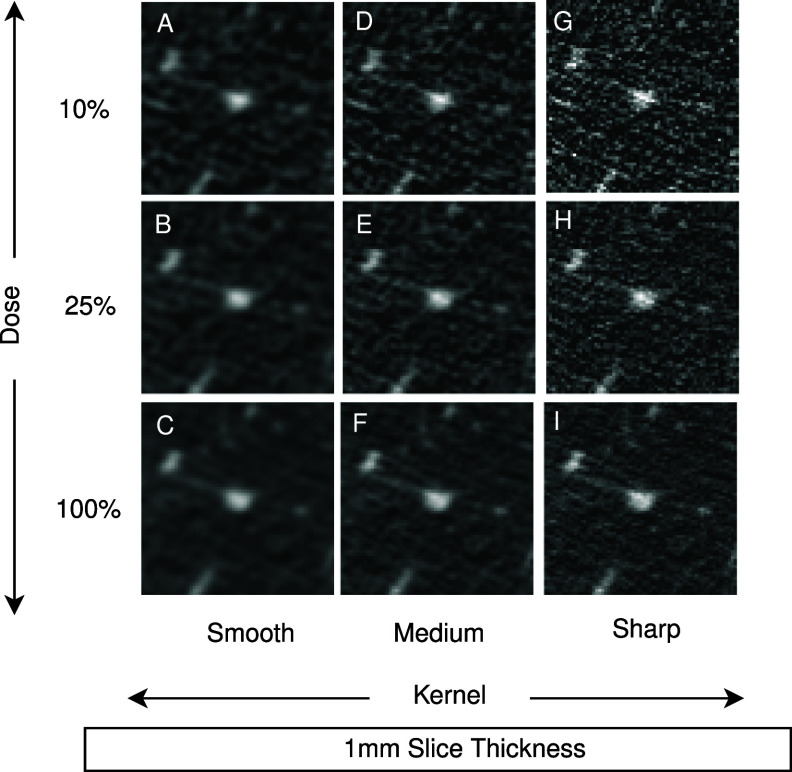
Examples of the same nodule under different image conditions. Condition A-I is generated from the acquired sinogram data by introducing noise to simulate reduced doses, which are then reconstructed using an open source weighted filtered back projection algorithm.

Using three reconstruction kernels (smooth, medium, sharp), which are comparable to Siemens B10f/B20f, B40f/B50f, and B60f kernels, original full-dose and simulated reduced dose projection data were reconstructed into images with dimensions of 512×512 with 1.0 mm slice thickness. The number of slices in the *z*-direction for each scan ranged from 258 to 390, with an average of 313 slices per scan. Figure 1 highlights differences in texture and appearance of a nodule across each of the nine reconstructed conditions. The reference image condition (see figure 1, condition F) for this analysis was set to be 100% dose, medium kernel, and 1.0 mm slice thickness, which is currently recommended for lung cancer screening (LUNG CANCER SCREENING CT 2019).

### 2.2. Study design

**Figure 2.**
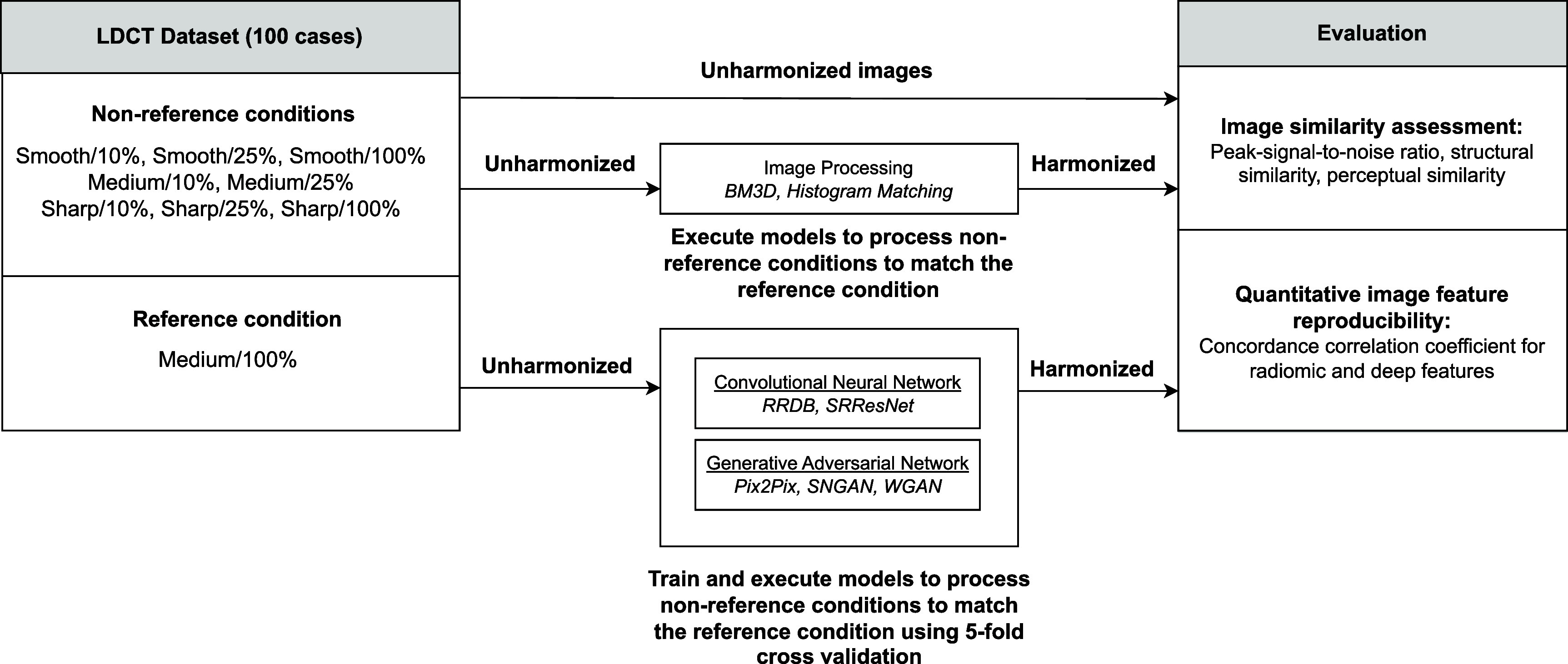
Flow diagram summarizing the study design. Using an in-house LDCT sinogram dataset that was reconstructed in multiple ways, we trained CNN and GAN-based harmonization methods using five-fold cross-validation. Each method was evaluated using two tasks, image similarity and quantitative image feature reproducibility.

Figure 2 summarizes the experimental design of this study. We implemented a set of harmonization techniques consisting of representative methods from three distinct groups: traditional image processing, convolutional neural networks (CNNs), and generative adversarial networks (GANs). The traditional image processing methods do not have any hyperparameters, and thus, no training data was used to parameterize these methods. The CNN and GAN-based methods were trained using a five-fold cross-validation approach (80–20 split for train and test per fold) on the in-house LDCT dataset. The objective was to map different non-reference image conditions to a reference image condition (defined here as medium kernel and 100% dose). Once training was done, we harmonized the non-reference conditions from each method on the test folds.

In the evaluation phase, we compared the efficacy of the harmonization methods at the image level and the feature level, with no harmonization as the baseline. Using the reference condition, we assessed image similarity using the peak signal-to-noise ratio (PSNR), structural similarity index measure (SSIM) and learned perceptual image patch similarity (LPIPS). We examined the reproducibility of quantitative image features (i.e. radiomic and deep features) across conditions using the concordance correlation coefficient (CCC).

### 2.3. Harmonization methods

We selected representative methods from three general types of harmonization approaches: traditional image processing, CNNs, and GANs. The focus on these three domains reflects their proven effectiveness in medical imaging, particularly for harmonization tasks (Nan *et al* 2022). Traditional image processing methods were included for their simplicity and established use in harmonization, providing a reliable benchmark. CNN-based methods were included due to their strength in capturing and reconstructing high-frequency details, which are often lost in low-dose CT scans. GANs were selected for their ability to generate realistic images and enhance the reproducibility of radiomic features. Methods within each domain were chosen to represent a range of approaches that have been validated across multiple datasets, and consistently deliver high-quality results in imaging tasks. Each domain is briefly introduced in the following sections.

#### 2.3.1. Traditional image processing

Block-matching and 3D Filtering (BM3D) reduces additive white Gaussian noise in images, which can potentially help dose-induced noise in CT images (Dabov *et al* 2007). The algorithm involves two main steps: thresholding and Wiener filtering, each with grouping, collaborative filtering, and aggregation stages. A noisy image is first divided into patches/blocks (*Q*), with a search window around each patch called the reference patch (*P*). Similar patches are identified within this window using the distance threshold $\tau^\mathrm{hard}$, defined by: $\mathcal{P}({P}) = {Q} : {d}({P},{Q}) \unicode{x2A7D} \tau^{\text{hard}}$, where ${d}({P},{Q})$ is the normalized quadratic distance between patches. These similar patches are stacked into a 3D group, which undergoes collaborative filtering to obtain an estimate for each patch that is aggregated and then refined using Wiener filtering (Lebrun 2012).

Histogram matching (HM) transforms an input scan so that its histogram matches a specified/reference histogram (Gonzalez and Woods 2007). For a discrete grayscale image, the probability of a pixel having a specific gray level *i* is given by $ P_x(i) = \frac{n_i}{n} $, where *n_i_* is the count of pixels with gray level *i*, *n* is the total number of pixels, and *L* is the total number of gray levels. The image’s histogram represents these probabilities. By calculating the cumulative distribution function for both the source and target images, a transformation is computed to map old intensity values to new ones, ensuring the target image’s pixel values closely match the source image’s pixel distribution.

#### 2.3.2. CNNs

We examined two different CNN-based methods both of which have demonstrated superior performance on multiple benchmark datasets, consistently outperforming other architectures in terms of sharpness and texture restoration. Ledig *et al* proposed a deep residual-based CNN for the task of estimating a high-resolution (HR) image from its low-resolution (LR) counterpart, i.e. single-image super-resolution (SISR) (2017). The model showed significant gains in perceptual quality on three public benchmark datasets. Their network architecture is a 16-block, deep super-resolution ResNet (SRResNet) with two convolutional layers within each residual block, followed by batch normalization layers and a parametric rectified linear unit as the activation function. This model was further improved upon by Wang *et al* who described two modifications to the SRResNet: removing all batch normalization layers and replacing the basic block with the proposed Residual-in-Residual Dense Block (RRDB) (2019).

In the original implementation, both the models were trained with a novel perceptual loss that uses high-level features from intermediate activation of a pre-trained VGG19 network (Simonyan and Zisserman 2014). However, for our harmonization purposes, loss computed on VGG19 features (pre-trained on *ImageNet*) would not be meaningful due to the significant differences in the image domains (Cheplygina *et al* 2019). Zhao *et al* experimented with several loss functions for image restoration and found that mean absolute error ($\mathcal{L}_1$) loss should be preferred over mean squared error ($\mathcal{L}_2$) since $\mathcal{L}_2$ is more sensitive to outliers and gets stuck easily in a local minimum (2017). We trained a single SRResNet and RRDB model using voxelwise $\mathcal{L}_1$ to optimize the distance between the reference and all other reconstructions. To accommodate the volumetric nature of the CT imaging data, we extended the 2D convolutional layers to 3D convolutions. This adaptation allows the models to better capture spatial relationships within 3D imaging volumes. Due to computational limitations, we reduced the number of residual blocks from 16 to 8 for both the SRResNet and RRDB models.

#### 2.3.3. GANs

GANs are implicit likelihood models that learn the statistical properties of a given dataset, synthesizing new examples drawn from an existing distribution of samples. A typical conditional GAN consists of a generator *G* that maps input scans to the reference scans and a discriminator *D* that learns to differentiate harmonized scans from reference samples. Isola *et al* proposed Pix2Pix, a conditional GAN designed for paired image-to-image translation tasks, which has been adapted for medical imaging applications (Isola *et al* 2017, Krishnan *et al* 2024). It employs an encoder-decoder architecture, typically a U-Net, that effectively preserves spatial information through skip connections while transforming the input image. To reduce computational complexity, we modified the number of downsampling layers in the U-Net to 4 while removing both batch normalization and the Tanh activation layer, as suggested by Lim *et al* in their image deblurring work (2017). Since batch normalization normalizes features, it can reduce the range flexibility, which is undesirable for our application.

Wei *et al* presented a spectral norm GAN (SNGAN), which uses an 8-block deep SRResNet-based generator and showed that it improved perceptual similarity in CT by 35% compared to a baseline CNN, while significantly reducing radiomic feature variability (2020). Wasserstein GAN (WGAN) is another GAN-based method designed to improve training stability and reduce mode collapse by using the Wasserstein distance instead of the Jensen-Shannon divergence, which is a common training objective for traditional GAN models (Arjovsky *et al* 2017). We use the WGAN with gradient penalty, a framework that imposes local regularization on the discriminator by enforcing the Wasserstein distance and satisfying the Lipschitz continuity constraint through this penalty (Gulrajani *et al* 2017).

We employed a VGG19-based discriminator to train all the GAN models, which demonstrated better convergence with two training objectives; the loss function for *G* combines adversarial loss and content loss ($\mathcal{L}_1$), as defined by: $ V_{G}(G,D) = - \alpha_1 \mathop{\mathbb{E}}_{x \sim p_{x}}[D_{\Theta}(G_W(x))] + \alpha_2 \mathop{\mathbb{E}}_{x \sim p_{x}, y \sim p_{y}} \|G_W(x) - y\|_1 $, where Θ and *W* are network parameters initialized using Kaiming initialization (He *et al* 2015). The discriminator’s loss function is given by: $ V_{D}(G,D) = \mathop{\mathbb{E}}_{y \sim p_{y}}[\textrm{min}(0, -1 + D_{\Theta}(y))] + \mathop{\mathbb{E}}_{x \sim p_{x}}[\textrm{min}(0, -1 - D_{\Theta}(G_W(x)))] $, which employs hinge loss to focus on difficult-to-classify samples. Similar to the approach taken for the CNN methods, we trained a single Pix2Pix, SNGAN and WGAN model with 3D convolutions to harmonize all different reconstructions in our dataset to the reference.

### 2.4. Experimental methods

#### 2.4.1. Experiment 1: image similarity assessment

Harmonized images were assessed using similarity metrics such as PSNR, SSIM, and LPIPS. PSNR and SSIM (equations (1) and (2)) quantify image quality degradation and are commonly used to measure local differences between the output (i.e. harmonized image) and a reference image,

\begin{equation*} \begin{aligned} \mathrm{PSNR}\left(y,\hat{y}\right) &amp; = 10\log_{10}\frac{{\mathrm{Max}\left(\hat{y}_{ij}, y_{ij}\right)^2}} {\frac{1}{mn} \sum_{i = 1}^m \sum_{j = 1}^n \left(\hat{y}_{ij}-y_{ij}\right)^2 }\\ \end{aligned}\end{equation*}

*y* refers to the reference and $\hat{y}$ refers to all other reconstructions. The number of rows and columns of pixels of the images is represented by *m* and *n*. *i* and *j* is the index of the corresponding row and column,

\begin{equation*} \begin{aligned} \mathrm{SSIM}\left(y,\hat{y}\right) &amp; = \frac{\left(2\mu_{\hat{y}}\mu_y + C_1\right) + \left(2 \sigma _{\hat{y}y} + C_2\right)} {\left(\mu_{\hat{y}}^2 + \mu_y^2+C_1\right) \left(\sigma_{\hat{y}}^2 + \sigma_y^2+C_2\right)} \\ \end{aligned}\end{equation*}

*C*_1_ and *C*_2_ are constants that stabilize the division. $\mu_{\hat{y}}$ and *µ*_*y*_ represents the mean of $\hat{y}$ and *y*, while $\sigma_{\hat{y}}$ and *σ*_*y*_ represents standard deviation. $\sigma _{\hat{y}y}$ is the covariance of $\hat{y}$ and *y*.

For PSNR and SSIM, higher values represent better reconstruction quality and closer similarity between images, respectively. A limitation of PSNR and SSIM is that they do not quantify content-dependent distortions and spatial shifts and thus cannot account for many nuances of visual perception (Kotevski and Mitrevski 2009). LPIPS, on the other hand, utilized a pre-trained VGG19 network to generate similarity scores from high-level feature space between two images and has been shown to correlate with human perception (Zhang *et al* 2018),

\begin{equation*} \begin{aligned} \mathrm{LPIPS}\left(y,\hat{y}\right) &amp; = {\sum_{l}\frac{1}{H_l,W_l}\sum_{h,w}||w_l\circ \left(y^{l}_{h,w} - \hat y^{l}_{h,w}\right)||^{2}_{2}}. \end{aligned}\end{equation*}

For each layer *l* in the VGG19 network, the height and width of the feature map are denoted as ${H_l}$ and ${W_l}$, respectively. These dimensions normalize the sum of the differences to account for the varying sizes of feature maps at different layers. The indices *h* and *w* represent the height and width positions within the feature map. The weight vector ${w_l}$ for layer *l* adjusts the contribution of each layer to the final similarity score, ensuring that the importance of different layers is appropriately balanced. $\mathcal{L}_2$ distance is then computed between the feature map activations of *y* and $\hat{y}$. A lower LPIPS value represents a closer distance to the reference image and thus indicates better image quality. For each scan in the test set, we calculate PSNR, SSIM, and LPIPS at the slice level, then average these values to obtain an overall measure for the scan. This process is repeated across 5 folds to generate a distribution of values for each image condition.

#### 2.4.2. Experiment 2: quantitative image feature reproducibility

We extracted radiomic and deep neural network-derived features as part of this analysis. Radiomic features have been used for a range of lung cancer-related tasks, including adenocarcinoma subtype classification, predicting tumor genotypes, assessing treatment response, and classifying other histologic subtypes (Li *et al* 2023). Similarly, deep features extracted from medical images have demonstrated significant utility in prognostic tasks, such as predicting patient outcomes, including survival rates and risk stratification (Chen *et al* 2024).

Prior to feature extraction, the voxel values were normalized to the range of [−1000, –500] Hounsfield Units, which is typically used for lung tissue analysis. For the radiomic features, Pyradiomics was used to extract 18 intensity and 68 texture features (van Griethuysen *et al* 2017). These features were calculated on the scans using a lung mask which was generated using an in-house segmentation model on the reference condition (Wang *et al* 2018). We did not extract shape features, as the mask remains constant across conditions; therefore, any shape features derived from it would not reflect variability between different reconstructions.

To extract deep features, we used a publicly shared deep learning-based indeterminate pulmonary nodules (IPNs) risk estimation model called DeepLungIPN (Gao *et al* 2022). The model is trained on a cohort from Vanderbilt University Medical Center and validated using three external datasets: The University of Colorado Denver, the University of Pittsburgh Medical Center, and the Detection of Early Cancer Among Military Personnel (DECAMP) study. It demonstrated superior performance compared to traditional risk assessment models, such as the Mayo and Brock models, in predicting lung cancer risk. The model comprises pre-trained modules for nodule detection and feature extraction. The feature extraction module identified features from the top five regions with the highest nodule confidence. These features were then fed into an attention-based multi-instance learning module to derive a five-element feature vector that characterizes the entire scan. We processed all exams through the model, including those without IPNs, such as cases with no nodules or nodules exceeding 30 mm in size, and then examined the variability in the resulting feature vector when using unharmonized and harmonized images.

CCC was used to compute an agreement score for radiomic and deep features across different reconstructions and the reference (Barnhart *et al* 2002),

\begin{equation*} \begin{aligned} \mathrm{CCC} &amp; = \frac{2 \rho \sigma_{\hat{y}} \sigma_y}{\sigma_{\hat{y}}^2 + \sigma_y^2 +\left(\mu_{\hat{y}} - \mu_y\right)^2} \\ \end{aligned}\end{equation*}

*ρ* is the Pearson correlation coefficient between reference features (*y*) and all other image condition features ($\hat{y}$). *µ*_*y*_ and $\mu_{\hat{y}}$ are the means, while *σ*_*y*_ and $\sigma_{\hat{y}}$ are the standard deviations of *y* and $\hat{y}$, respectively. We divided the CCC score into three different agreement levels. Based on a previously validated interpretation of this metric, a CCC greater than 0.9 is considered as having a ‘good’ agreement, whereas between 0.8 and 0.9, a ‘moderate’ agreement, and below 0.8, a ‘poor’ agreement (Yang *et al* 2016). For each respective radiomic and deep feature, we obtained a set of values from all scans in the test set. We then computed the CCC for these values. This process was repeated across five folds to generate a distribution of CCC values for each image condition.

## 3. Results

### 3.1. Experiment 1: image similarity assessment

**Figure 3.**
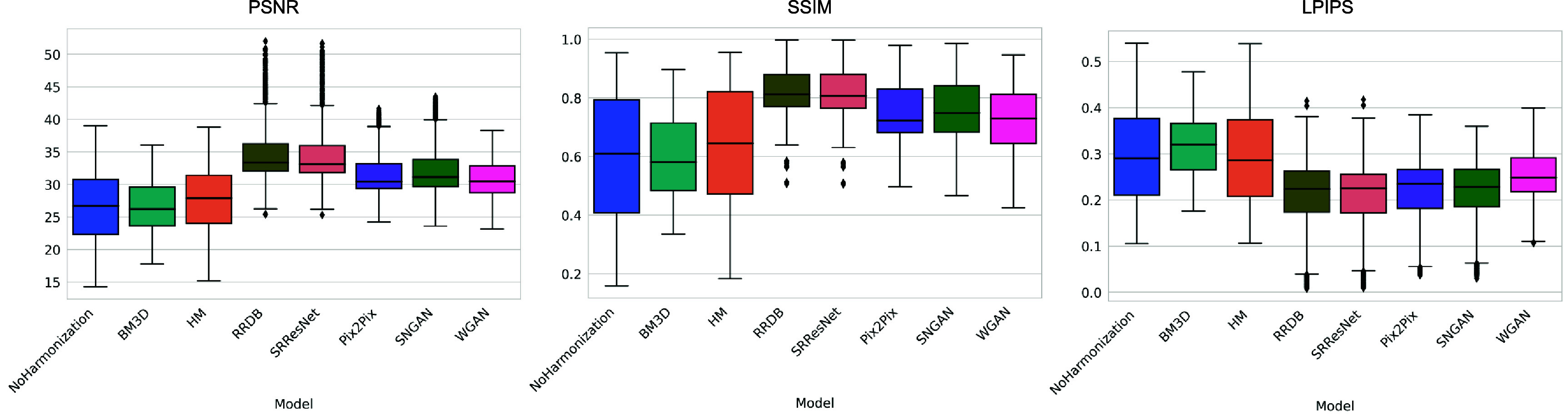
Box plots of the similarity metrics across harmonization methods: PSNR (left), SSIM (middle), and LPIPS (right). $\uparrow$ Higher values indicate better performance for PSNR and SSIM; $\downarrow$ Lower values indicate better performance for LPIPS.

**Figure 4.**
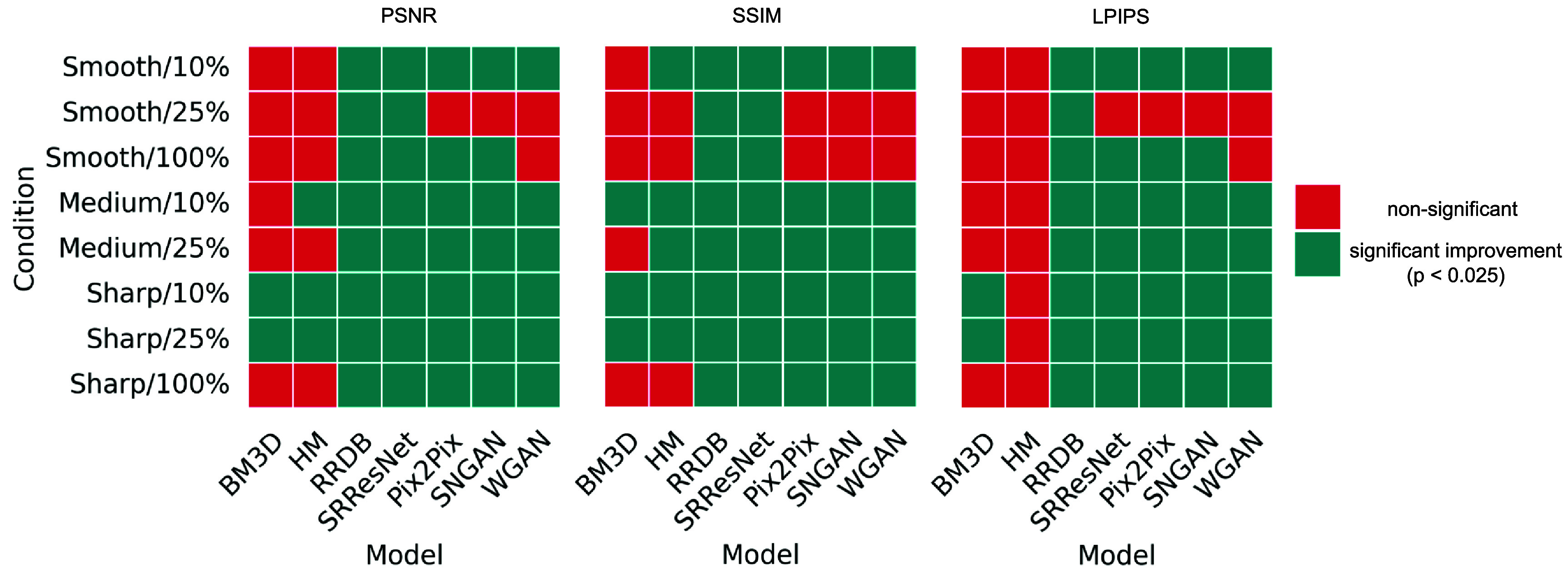
Differences in harmonization methods based on image similarity metrics. For each image condition, metrics were computed for harmonized and unharmonized scans, with statistical significance assessed using the Wilcoxon signed-rank test with Benjamini–Hochberg correction.

The box plot shown in figure 3 summarizes the distribution of PSNR, SSIM, and LPIPS, which are computed with respect to the reference condition; the box plots are computed using all non-reference conditions. The baseline unharmonized metrics were 26.678 ± 4.922 (mean ± 95% confidence interval) for PSNR, 0.593 ± 0.199 for SSIM, and 0.295 ± 0.100 for LPIPS. As summarized in figure 4, we conducted a one-tailed Wilcoxon signed-rank test with Benjamini–Hochberg correction to check for a significant increase in PSNR and SSIM and a decrease in LPIPS after harmonization, using a significance level of 0.025.

Significant statistical improvements were observed after harmonization. Within the image processing methods, BM3D consistently resulted in a lower image similarity compared to HM (i.e. 26.551 ± 3.239 vs 27.742 ± 4.511 in PSNR, 0.601 ± 0.123 vs 0.627 ± 0.186 in SSIM, and 0.319 ± 0.057 vs 0.292 ± 0.100 in LPIPS). Among the CNN methods, RRDB and SRResNet yielded comparable performance, with PSNR, SSIM, and LPIPS values of 35.579 ± 4.716 vs 35.467 ± 4.887, 0.832 ± 0.078 vs 0.830 ± 0.081, and 0.194 ± 0.084 vs 0.192 ± 0.082, respectively. Within the GAN methods, Pix2Pix and SNGAN consistently outperformed WGAN, with PSNR, SSIM, and LPIPS values of 32.079 ± 3.456 vs 32.761 ± 3.887 vs 30.910 ± 2.346, 0.760 ± 0.096 vs 0.772 ± 0.102 vs 0.731 ± 0.096, and 0.210 ± 0.070 vs 0.205 ± 0.072 vs 0.247 ± 0.048, respectively.

**Figure 5.**
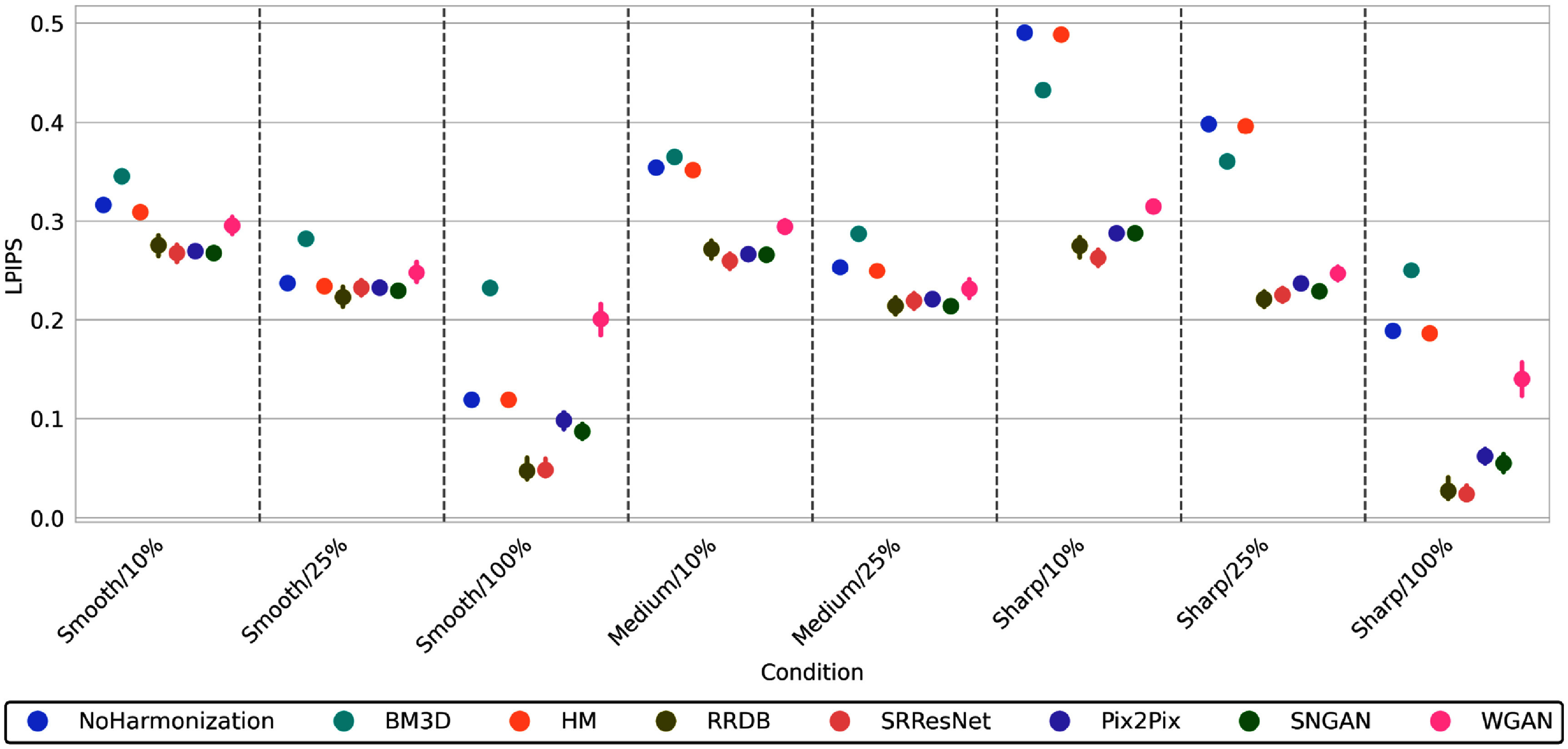
Point plot summarizing the mean LPIPS for each model. 95% confidence intervals are provided for models that underwent five-fold cross-validation with error bars computed across each fold, stratified by image condition.

To better understand the impact of these methods across variable conditions, figure 5 summarizes the mean LPIPS score, with lower scores indicating better image similarity with the reference. Without harmonization, Smooth/100% achieved the best LPIPS score (i.e. 0.120 ± 0.003), while Sharp/10% achieved the worst (i.e. 0.490 ± 0.005) across all conditions. As the radiation dose level increased within each reconstruction kernel (e.g. comparing Smooth/10% to Smooth/25% and Smooth/100%), LPIPS decreased. This decrement was smaller when increasing the dose from 10% to 25% (decrement of 0.080, 0.101, and 0.091 for smooth, medium, and sharp kernels, respectively) compared to increasing it from 25% to 100% (decrement of 0.117 and 0.210 for smooth and sharp kernels, respectively). As the kernel varied within each dose level (e.g. comparing Smooth/10% to Medium/10% and Sharp/10%), LPIPS increased (i.e. decrease in image similarity). This increment was smaller when transitioning from smooth to medium kernel (0.037 at 10% dose and 0.016 at 25% dose) compared to transitioning from either medium to sharp kernel (0.136 at 10% dose and 0.146 at 25% dose) or from smooth to sharp kernel (0.173 at 10%, 0.162 at 25%, and 0.069 at 100% dose).

**Figure 6.**
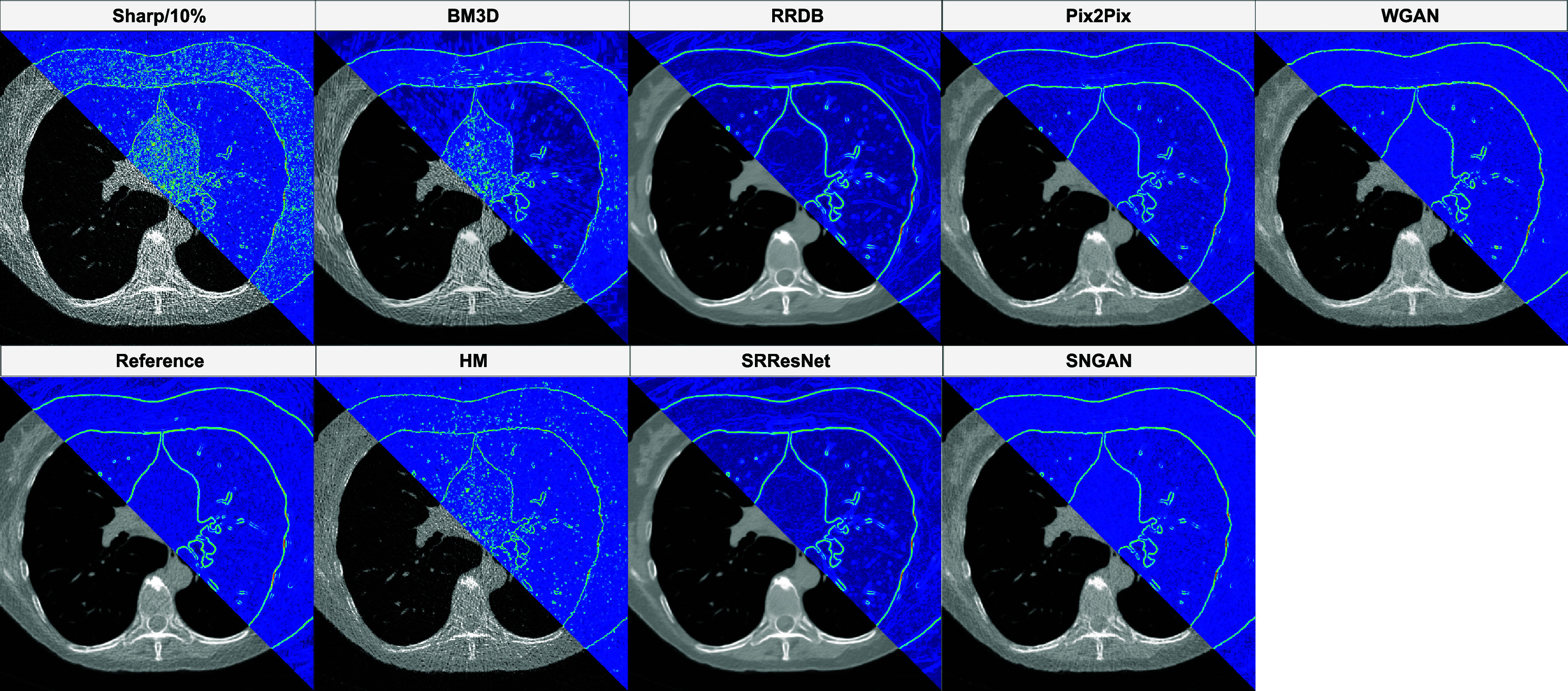
Images after harmonization from each of the different methods. Top left: No harmonization under Sharp/10%; Bottom left: Reference condition. The composite images were generated by applying a Sobel filter to highlight the noise component of each image.

Traditional image processing-based methods did not have much of an impact on LPIPS; BM3D resulted in worsened LPIPS across all conditions, except for a significant decrease in sharp kernel scans at the 10% and 25% dose levels (see figure 4). HM did not lead to a significant decrease in LPIPS for any condition. A qualitative assessment of the harmonized images, as shown in figure 6, revealed that image processing techniques were unable to effectively mitigate increased noise in lower dose scans. Both the CNN and GAN methods significantly reduced LPIPS across a majority of conditions; These methods were most effective on Sharp/10% (average decrement of 0.221 for CNNs and 0.193 for GANs). SRResNet, Pix2Pix, and SNGAN were least effective on Smooth/25%, where no significant decrease in LPIPS was observed, while WGAN was the worst-performing method for Smooth/25% and Smooth/100%, where an increase in LPIPS was observed. Comparing all methods, CNN-based methods achieved the best combination of PSNR, SSIM, and LPIPS, with RRDB achieving the best performance across all metrics and image conditions.

### 3.2. Experiment 2: quantitative image feature reproducibility

**Figure 7.**
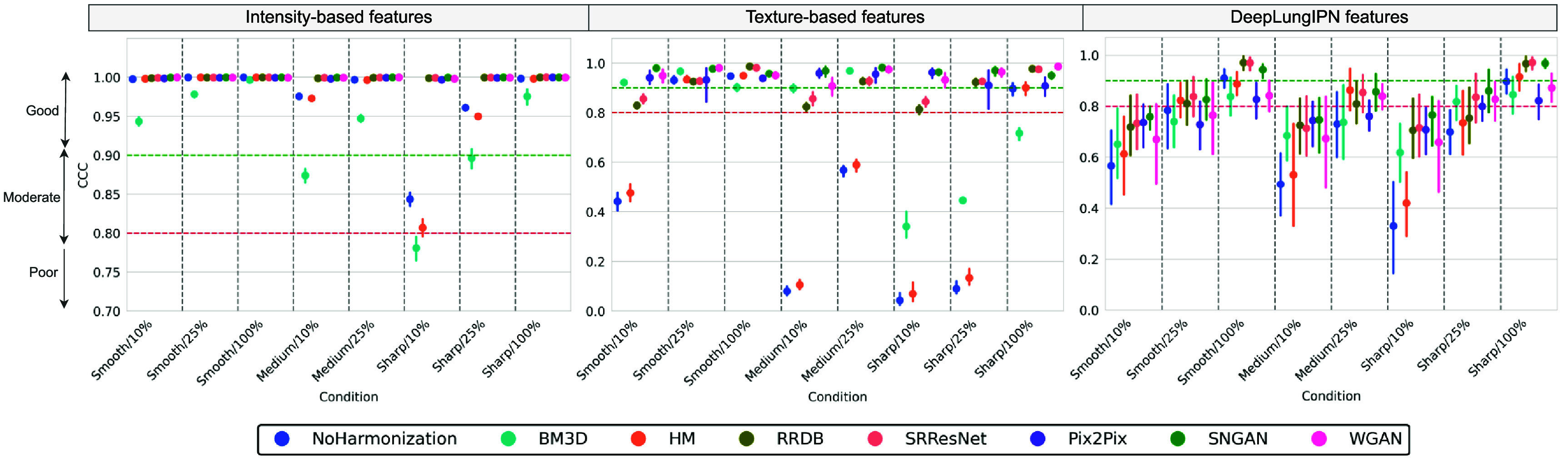
Point plots summarizing the mean CCC. 95% confidence intervals are provided for models that underwent five-fold cross-validation with error bars computed across each fold, stratified by image condition and radiomic/deep features.

Figure 7 summarizes the mean CCC score after harmonization for each condition across two categories of radiomic features (intensity and texture) and deep features. Intensity-based features, which reflected characteristics such as tissue density, voxel intensity, and contrast enhancement, resulted in a CCC of 0.972 ± 0.045 (mean ± 95% confidence interval) across conditions without harmonization. Good agreement was observed across all conditions except Sharp/10% (CCC of 0.844 ± 0.014). Harmonizing this condition using CNN and GAN-based methods improved the agreement level to ‘good’. Additionally, in cases where there was a ‘good’ agreement with no harmonization (e.g. Medium/10% and Sharp/25%), image processing-based harmonization such as BM3D reduced the CCC score.

Texture-based features exhibited a greater degree of variability (i.e. CCC of 0.500 ± 0.332) compared to intensity features. Three out of the eight conditions (i.e. Smooth/25%, Smooth/100%, and Sharp/100%) showed ‘good’ agreement with the reference with no harmonization; Sharp/10% had the lowest CCC (i.e. 0.043 ± 0.039) while Smooth/100% had the highest (i.e. 0.948 ± 0.012). As the dose level increased within each reconstruction kernel, CCC also increased, with smooth and medium kernels showing a larger increase from 10% to 25% (average increment of 0.488) than from 25% to 100% (average increment of 0.017), while the sharp kernel had a smaller increase from 10% to 25% (0.048) but a significantly larger increase from 25% to 100% (0.806). As the kernel varied within each dose level, CCC decreased, with a larger drop from smooth to medium (0.362) compared to from medium to sharp (0.037) at 10% dose, an incremental decrease from smooth to medium and then to sharp at 25% (decrement of 0.363 and 0.477, respectively), and minimal impact at 100% (decrement of 0.051).

Deep features resulted in a CCC of 0.676 ± 0.168 across conditions with no harmonization, with confidence intervals showing higher variation within folds; on average, the standard error across conditions for deep and radiomic features was 0.174 and 0.034, respectively. Only the conditions at 100% dose level (i.e. Smooth/100% and Sharp/100%) resulted in ‘good’ agreement, whereas the remaining six conditions had ‘poor’ agreement. The trends observed in deep features with no harmonization were consistent with what was observed in texture features; Sharp/10% had the lowest CCC (i.e. 0.330 ± 0.288), while Smooth/100% had the highest (i.e. 0.910 ± 0.060). As the dose level increased within each reconstruction kernel, CCC increased, with a larger increase from 10% to 25% (0.218 for smooth, 0.237 for medium, and 0.369 for sharp) than from 25% to 100% (0.126 for smooth and 0.198 for sharp). Conversely, varying the kernel (other than smooth) within each dose level resulted in a decrease in CCC. At the 10% dose level, changing the kernel from smooth to medium resulted in a smaller decrease (0.072) than from medium to sharp (0.164), while at 25%, the shift from smooth to medium to sharp gradually reduced CCC (decrements of 0.053 and 0.032, respectively), and at 100%, there was no significant impact (decrement of 0.013).

**Figure 8.**
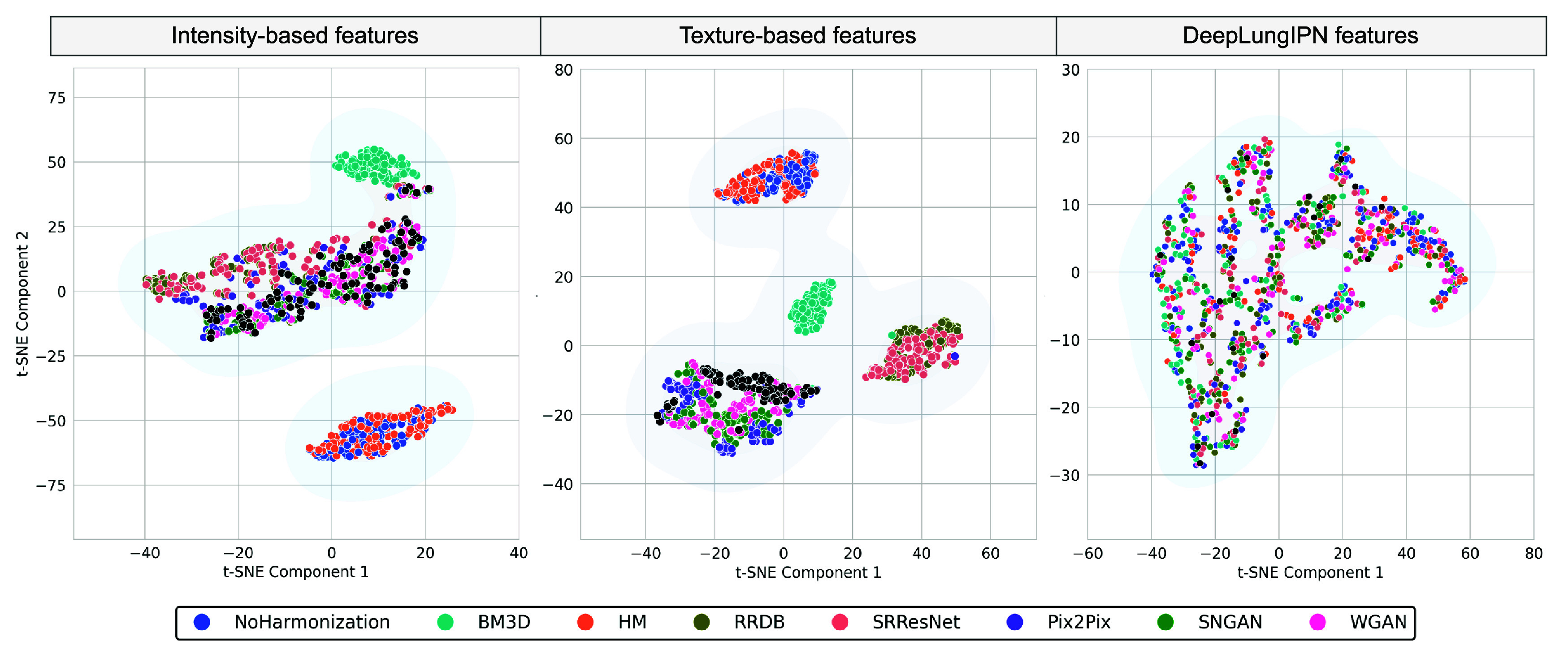
t-SNE plot showing the alignment of unharmonized and harmonized features with the reference (marked in black) under the Sharp/10% image condition for two categories of radiomic features and a set of deep features.

**Figure 9.**
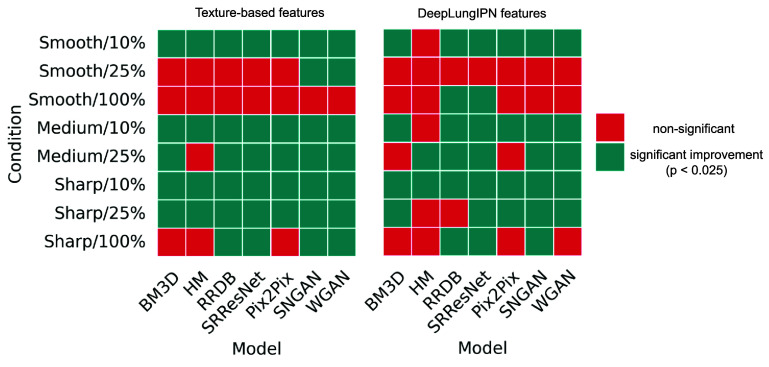
Differences in harmonization based on a comparison of radiomic and deep features. For each image condition, metrics were computed for harmonized and unharmonized scans, with statistical significance assessed using the Wilcoxon signed-rank test with Benjamini–Hochberg correction.

The condition that consistently performed worst across different categories of features was Sharp/10%. For this condition, we summarized the alignment of features with the reference before and after harmonization in figure 8. The t-SNE plot showed that intensity and texture features formed distinct clusters and were well-separated in the high-dimensional space before and after harmonization. For intensity features, both CNN and GAN-based methods effectively aligned unharmonized features with the reference, while for texture features, GANs aligned the features better than CNNs. For deep features, we did not see a clear separation of clusters before and after harmonization, indicating high complexity and non-linearity in features. As summarized in figure 9, we performed a one-tailed Wilcoxon signed-rank test using a significance level of 0.025 to evaluate the significant increase in CCC after harmonization for radiomic and deep features, where the highest variability across conditions is observed.

BM3D harmonization significantly increased CCC for texture features across all kernels at 10% and 25% dose levels, except for Smooth/25%. It improved the agreement level for three conditions (i.e. Smooth/10%, Medium/10%, and Medium/25%), with Medium/10% showing the most significant increment of 0.819. It also lowered the CCC level for sharp/100% to ‘poor’, which was initially at a ‘good’ agreement without harmonization. Even though HM showed significant improvements in smooth and medium kernel scans at 10% dose and in sharp kernel scans at 10% and 25% doses, it resulted in no change in agreement level on any condition. For deep features, BM3D showed the most notable improvement in the Sharp/10%, with an increment of 0.288. HM performed poorly in improving deep features CCC as it did for radiomic features, except for Medium/25%, where a significant increment of 0.133 was observed.

SRResNet and RRDB significantly increased CCC for texture features across all conditions, except for Smooth/25% and Smooth/100%, where the CCC was already in ‘good’ agreement with no harmonization. Both the models changed the agreement level from ‘poor’ to ‘good’ at 25% dose levels (i.e. Medium/25% and Sharp/25%), and to ‘moderate’ at 10% dose levels (i.e. Smooth/10%, Medium/10% and Sharp/10%). They were most effective on Sharp/25%, with an average increment of 0.833. For deep features, RRDB failed to significantly improve CCC for Smooth/25% and Sharp/25%, whereas SRResNet failed only for Smooth/25%. Unlike what was observed with radiomic features, both RRDB and SRResNet were most effective on Sharp/10%, with an average increment in CCC of 0.380.

Pix2Pix, SNGAN, and WGAN resulted in a significant increase in CCC for texture features, improving the level of agreement to ‘good’ across all five conditions, which was ‘poor’ before harmonization. Pix2Pix showed a greater degree of variability in CCC across folds compared to SNGAN and WGAN for a majority of conditions. The GAN models were most effective on Sharp/10%, with an average increment in CCC of 0.909. For deep features, SNGAN and WGAN failed to significantly improve CCC for Smooth/25%, while Pix2Pix also failed for Medium/25%. They were most effective on Sharp/10%, with an average increment in CCC of 0.380, mirroring similar trends observed in radiomic feature agreement.

None of the CNN and GAN-based harmonization methods improved the agreement level for deep features to ‘good’ for any conditions that were ‘poor’ or ‘moderate’ with no harmonization. In summary, SNGAN yielded a CCC of 0.969 ± 0.009 for texture-based radiomic features and 0.841 ± 0.070 for deep features across conditions, surpassing all other CNN and image processing methods.

## 4. Discussion

The study underscores that the choice of data harmonization technique is driven by image condition and downstream application, as CT parameters uniquely influence downstream tasks we aim to optimize. Conclusions drawn from one type of experiment (e.g. image similarity) might not yield similar trends in other experiments (e.g. quantitative image feature reproducibility). While increasing radiation dose generally enhanced image similarity and improved image feature agreement, the extent of changes in LPIPS and CCC varied depending on the reconstruction kernel. For instance, in terms of image similarity, as the dose level increased, smooth and medium kernel scans exhibited a relatively smaller degree of improvement in LPIPS compared to sharp kernel scans. This indicated that sharp kernel scans benefit significantly with dose level increments, especially at higher doses (i.e. 100%). However, in the case of radiomic feature agreement, smooth and medium kernel scans demonstrated greater improvement in CCC at lower dose levels (from 10% to 25%), while the sharp kernel exhibited greater improvement only at the higher dose (from 25% to 100%).

In the case of deep feature agreement, across all kernels, scans at lower doses showed a greater degree of improvement compared to higher doses. The change in kernel parameter also had a distinct impact on each metric at lower dose levels. Image similarity was worse, as reflected by an increased LPIPS metric, when changing the kernel from smooth to medium and even more so when changing from medium to sharp. Radiomic feature agreement experienced a larger drop in CCC when transitioning from smooth to medium rather than from medium to sharp. Conversely, for deep feature agreement, the change from smooth to medium kernel showed a smaller decrease in CCC compared to the more significant drop observed when transitioning from medium to sharp. For both radiomic and deep features, the observed trends diminish as the dose level increases to 100%. Thus, kernel variation impacts CCC more significantly at lower dose levels than at higher doses.

With no harmonization involved, a higher dose level and smooth kernel achieve the best perceptual similarity, as measured by LPIPS, and feature agreement, as measured by CCC. A discernible pattern exists in how specific parameters interact across different analyses. Some conditions do not require harmonization. For instance, Smooth/100% was perceptually closest to the reference, and the CCC score for this condition was the highest for both radiomic and deep features without any harmonization. Conversely, certain conditions, especially those at very low doses (i.e. less than 25%), greatly benefit from harmonization. For example, prior to harmonization, Sharp/10% consistently resulted in the worst image similarity and the lowest CCC scores for radiomic and deep features. Applying harmonization significantly improved the performance outcomes on both metrics.

Certain conditions are particularly challenging to harmonize. For instance, all methods failed on Smooth/25% when it came to improving deep feature agreement to ‘good,’ whereas only RRDB improved the perceptual similarity score for this condition. These results highlight the need for targeted application of harmonization methods. One-size-fits-all approaches may not be effective, and methods should be customized based on the specific imaging conditions or characteristics of the data.

Specific sets of features are more robust to variations. For example, intensity-based features remain stable across reconstructions as long as the images are not extremely noisy (i.e. less than 25% dose). Normalizing voxel values can effectively mitigate these variations. In contrast, texture-based radiomic and deep features exhibited a greater degree of variability across conditions. Across different folds of the five-fold cross-validation, deep features showed significantly higher variations than texture-based features because deep models capture more nuanced patterns in images. Even though harmonization aided in standardizing and generating ‘good’ radiomic features, it seemed to struggle to achieve a ‘good’ agreement level for deep features. Deep features varied significantly depending on input data. Regardless, harmonization enhances the reproducibility of image features, which is crucial for downstream machine learning applications, ultimately leading to more accurate and reliable outcomes.

Harmonization is not universally beneficial. For instance, BM3D consistently showed significant improvement in PSNR, SSIM, and LPIPS for sharp kernel scans at low doses (i.e. 10% and 25%), although it lowered the CCC for intensity-based features on these scans. Depending on the evaluation metric we prioritize, certain methods should take precedence. According to LPIPS measurements, traditional image processing methods such as HM and BM3D performed suboptimally compared to CNN and GAN approaches. These traditional methods lack the trainable parameters necessary to adapt to the specific noise characteristics of low-dose images. In contrast, CNN-based methods outperformed GANs in improving image similarity. However, for quantitative image feature reproducibility, GANs proved more effective than CNNs. This distinction underscores the difference between harmonization aimed at improving visual interpretation and harmonization aimed at enhancing the performance of machine learning models that leverage quantitative features as inputs.

This study had several notable limitations. First, the deep learning models were trained on a relatively small set of cases and evaluated using limited image conditions and CT parameters. However, the availability of real-world sinogram data, allowing us to generate a wide range of image conditions, is limited. While we selected a wide range of doses, including extreme conditions such as 10% low-dose, to evaluate the capabilities of harmonization techniques, these conditions are not observed in practice. We plan to generate more cases on a much broader set of image conditions (e.g. introduce 50% dose level, different slice thickness) using our CT reconstruction pipeline to generate additional conditions that match what may be observed in clinical practice. Second, the implemented harmonization methods were based on the authors’ original implementation, and this work did not optimize them. For instance, Hasan *et al* proposed an improved version of BM3D where the authors attempted to enhance the Wiener filter by maximizing the SSIM between the true and estimated images instead of minimizing the mean square error between them (Hasan and El-Sakka 2018).

Third, this analysis did not address other sources of variability (e.g. measurement differences, variation in deep feature extraction models, or differences across imaging modalities and patient populations). For instance, in the case of the deep feature agreement analysis, conclusions regarding feature reproducibility could differ if a model other than DeepLungIPN was used. Since the model is optimized for cases with IPNs, and our dataset contains relatively few exams with IPNs, the generalizability of the deep feature variability analysis to broader clinical scenarios is limited. In addition, the harmonization results are tied to a specific modality (i.e. CT) and lung cancer screening patient population data. Their generalizability to other imaging modalities, such as MRI or PET, may be limited due to differences in acquisition techniques and image characteristics; for example, MRI’s sensitivity to soft tissues and PET’s focus on metabolic activity present unique challenges for harmonization compared to CT. Variability in patient populations, including factors such as age, comorbidities, and anatomical differences, may necessitate model adjustments to maintain performance across diverse groups.

Understanding the efficacy of harmonization under different imaging conditions for various downstream analyses enables us to develop models that can adapt to and perform consistently across diverse imaging settings. This ensures reliable outcomes in various scenarios where automated computer-aided detection/diagnosis models are integrated into clinical workflows. Our future work will focus on establishing whether the observed improvements in perceptual similarity and reproducibility metrics from harmonization in CT translate to actual diagnostic performance gains. In clinical settings, diagnostic relevance often depends on subtle textural and morphological cues that general image quality metrics may not fully capture, and the ultimate validation of any harmonization technique lies in its ability to enhance diagnostic accuracy and patient outcomes.

## 5. Conclusion

A variety of methods exists for image harmonization, ranging from traditional image processing to image synthesis methods. The effectiveness of these approaches in mitigating differences in CT scans to achieve reliable performance in downstream tasks has not been comprehensively characterized. This study provides a systematic approach to characterize the effect of CT parameters and image harmonization techniques, providing a basis for deciding whether harmonization is needed and what harmonization method to use, depending on the input CT scans and the task.

## Data Availability

The data cannot be made publicly available upon publication due to legal restrictions preventing unrestricted public distribution. The data that support the findings of this study are available upon reasonable request from the authors.
